# Nonspecific membrane-matrix interactions influence diffusivity of lipid vesicles in hydrogels

**DOI:** 10.1016/j.bpj.2024.02.005

**Published:** 2024-02-07

**Authors:** Nicky W. Tam, Otto Schullian, Amaia Cipitria, Rumiana Dimova

**Affiliations:** 1 Max Planck Institute of Colloids and Interfaces, Science Park Golm, Potsdam, Germany; 2 Free University of Berlin, Department of Physics, Berlin, Germany; 3 Group of Bioengineering in Regeneration and Cancer, Biogipuzkoa Health Research Institute, San Sebastián, Spain; 4 IKERBASQUE, Basque Foundation for Science, Bilbao, Spain

## Abstract

The diffusion of extracellular vesicles and liposomes in vivo is affected by different tissue environmental conditions and is of great interest in the development of liposome-based therapeutics and drug-delivery systems. Here, we use a bottom-up biomimetic approach to better isolate and study steric and electrostatic interactions and their influence on the diffusivity of synthetic large unilamellar vesicles in hydrogel environments. Single-particle tracking of these extracellular vesicle-like particles in agarose hydrogels as an extracellular matrix model shows that membrane deformability and surface charge affect the hydrogel pore spaces that vesicles have access to, which determines overall diffusivity. Moreover, we show that passivation of vesicles with PEGylated lipids, as often used in drug-delivery systems, enhances diffusivity, but that this effect cannot be fully explained with electrostatic interactions alone. Finally, we compare our experimental findings with existing computational and theoretical work in the field to help explain the nonspecific interactions between diffusing particles and gel matrix environments.

## Significance

The diffusion of nanoparticles in human tissues is dependent on interactions with the surrounding environment. This has wide implications for the development of nanoparticle-based therapeutics and drug-delivery systems. Studying these interactions in human tissues and even in model hydrogels composed of reconstituted tissue components can be hampered by the complexity of these materials. By using a bio-inert hydrogel such as agarose, we remove the influence of specific biochemical interactions, allowing the study of how particle diffusion can be tuned with simple material properties such as charge and rigidity. Taking advantage of these nonspecific interactions, nanoparticles could one day be engineered to target specific organs by optimizing diffusion in certain tissue environments or retention and immobilization in others.

## Introduction

Large unilamellar vesicles (LUVs), or liposomes, are phospholipid structures 100–1000 nm in diameter that are often used as a minimal model of cell-derived extracellular vesicles (EVs). Their application in drug-delivery systems takes advantage of the structure and function of their in vivo counterparts, as facilitators of intercellular transport (1,2) to shield their payloads from the external tissue environment and mediate their transport to and uptake by target cells (3,4). Despite the rising interest in using such lipid nanoparticle systems (for example, to deliver anti-cancer therapeutics (5,6) or as carriers of immunogenic materials in vaccine formulations (7), there are few data on how nanoparticle mobility and transport within tissues is affected by different tissue environmental conditions and membrane material properties.

A 2009 study by Lieleg et al. (8) showed that hydrogel materials derived from extracellular matrix (ECM) can act as an electrostatic filter, sequestering charged nanoparticles but allowing neutral particles to pass through unimpeded. Later, Yu et al. (9) and Lenzini et al. (10) found that the deformability of lipid vesicles, modulated by the lipid composition of the membrane and by the presence of water-permissive channel proteins, respectively, can influence their access through hydrogel pore spaces and thus their movement and transport. Other work, including studies on rigid polymeric nanoparticles diffusing in polymer solutions (11,12), in colloidal mucin suspensions (13), and in hydrogels (14,15,16,17), have also investigated the various ways in which charge and steric interactions affect the dynamics of nondeforming particles. Clearly, particle diffusion is affected by a diverse range of biophysical factors and of particular interest is the way these factors might interact. For example, recent theoretical work suggests that particle diffusability is a balancing act between particle deformability and particle-matrix adhesion.(18) When particles or vesicles are subjected to surface modifications, such as PEGylation (8,13,19,20,21,22,23) or the inclusion of more complex molecules, their surface interactions with the surrounding medium must also be taken into account. It is not difficult to imagine, then, that the combined effects of such interactions can give rise to the complex distribution patterns of vesicles observed in vivo (24).

To systematically study how different material properties of lipid vesicles and ECM-like hydrogels can influence vesicle diffusion, we use single-particle tracking (25) to study the diffusion of synthetic LUVs embedded in agarose. Agarose is a polysaccharide polymer from red algae that undergoes thermo-reversible gelation via noncovalent hydrogen bonding (26,27). Although much simpler in chemical composition than the diverse molecules found in human ECM, agarose provides greater control over material properties. Stiffness and porosity of agarose gels, for example, can be found in a comparable range to human tissues such as brain or cartilage and can be controlled with concentration and gelation conditions (28,29,30). Agarose is also a relevant material used in a number of different biomedical applications (28,31,32), including in three-dimensional cell culture platforms (33,34) and as components of composite materials for tissue engineering (28,35,36,37). Most importantly, agarose is bio-inert (33), allowing for the investigation of nonspecific steric and electrostatic interactions without the influence of specific biochemical interactions that may be present with reconstituted ECM materials or mucin suspensions. By using this biomimetic system, we aim to better understand the biophysical mechanisms that govern the diffusion of extracellular-like vesicles or drug carriers in tissue-like materials. We also aim to directly compare deformable vesicles with similarly sized rigid nanoparticles and particles with surface modifications to better tease apart how particle deformability and surface interactions contribute to overall particle diffusion and dynamics. Altogether, these results could one day lead to more efficient targeting and delivery of lipid nanoparticle-based therapeutics and vaccine delivery (3,5,6,7,38).

## Materials and methods

### LUV production and characterization

Lipid stocks dissolved in chloroform (Avanti Polar Lipids, Alabaster, AL, USA) were used to prepare mixtures containing 4 mM 1,2-dioleoyl-sn-glycero-3-phosphocholine (DOPC) as a base solution. Negatively charged LUVs were made with a 2:1 molar ratio mixture of DOPC and 1,2-dioleoyl-sn-glycero-3-phospho-L-serine (DOPS), whereas positively charged LUVs were made with a 2:1 molar ratio mixture of DOPC and 1,2-dioleoyl-3-trimethylammonium-propane (DOTAP). PEGylated lipids were used to passivate vesicles for diffusion in hydrogels. At room temperature, all lipids are above their main phase transition temperatures and no demixing in the membrane is expected to occur. For PEGylated LUVs, additions of 1 mol % or 10 mol % 1,2-distearoyl-sn-glycero-3-phosphoethanolamine-N-[methoxy(polyethylene glycol)-1000], -2000], or -5000] (DSPE-mPEG1K, DSPE-mPEG2K, and DSPE-mPEG5K, respectively) were added to base solutions of DOPC or DOPC/DOTAP. Fluorescent visualization was facilitated by the addition of 0.2 mol % DiIC_18_ (5) (Thermo Fisher Scientific, Waltham, MA, USA; 1,1′-dioctadecyl-3,3,3′,3′-tetramethylindodicarbocyanine, 4-chlorobenzenesulfonate salt).

LUVs were produced by first spreading a thin layer of a lipid mixture inside a glass vial and drying under vacuum for 1.5 h. Next, the lipid film was hydrated with phosphate-buffered saline (PBS; tablets for 200-mL solutions from Sigma-Aldrich, St. Louis, MO, USA) and vortexed for 30 min to produce multilamellar lipid structures. The resulting solution was then extruded with a Mini Extruder (Avanti Polar Lipids, Alabaster, AL, USA), 21 passes each through a 200- and 100-nm polycarbonate Nuclepore Track-Etched Membrane (Sigma-Aldrich, St. Louis, MO, USA).

Size distribution and zeta potential of particles were measured using a Malvern Instruments Nano-ZS Zetasizer equipped with a 632.8-nm 4-mW HeNe laser to ensure sample consistency. Samples in disposable folded capillary cells (DTS1070; Malvern Panalytical, Malvern, UK) were analyzed with dynamic light scattering (DLS) at a scattering angle of 173° to determine size distribution before determination of zeta potential. All particles were measured in high-salt buffer conditions resulting in electrostatic screening, so zeta potential values are used to illustrate relative differences in surface charge rather than absolute charge.

### Fluctuation analysis

To probe how the bending rigidity of lipid membranes changes with the presence of PEGylated lipids, we used fluctuation analysis on giant unilamellar vesicles (GUVs) (39,40). GUVs were made using the gel-assisted swelling method (41,42) (see section S1 in the Supporting Material). Briefly, 20 *μ*L of 5% w/v solution of polyvinyl alcohol (fully hydrolyzed, molecular weight, 145,000 Da; Merck Group, Darmstadt, Germany) in water with 50 mM sucrose was spread onto a 2-cm by 5-cm area corresponding to the dimensions of a rectangular, 2-mm-thick Teflon spacer and allowed to dry completely in an oven at 50°C. Next, a thin 15-*μ*L layer of 4 mM lipid mixture dissolved in chloroform was spread on top of the polyvinyl alcohol layer and dried in a vacuum for 1.5 h. The slide was then assembled into a sandwich with another glass slide and a Teflon spacer in the middle, held together with binder clips (Fig. S1). The lipid layer was hydrated for 30 min with 2 mL of PBS + 50 mM sucrose (345 mOsm/kg). The sucrose was necessary to help with the swelling process and to generate a sugar gradient that would later aid in visualizing the GUVs. GUVs were harvested and diluted 1:1 in a solution of PBS + 100 mM glucose (394 mOsm/kg) to slightly deflate the GUVs and were visualized under phase contrast with a 40× objective on a Zeiss AXIO Observer.D1 microscope. Image sequences of 3000 frames were recorded with a pco.edge sCMOS camera (Excelitas Technologies, Waltham, MA, USA) at 25 frames per second (fps) with 200-*μ*s exposure. Fluctuation analysis software (40) computed the bending rigidity based on the Fourier decomposition of thermally driven membrane fluctuations into spherical modes. Fluctuation analysis as well as all other experiments were conducted at room temperature, approximately 23°C.

### Preparation and characterization of agarose gels

Stock solutions of 2% w/v low-gelling-temperature agarose (BioReagent, for molecular biology; Sigma-Aldrich, St. Louis, MO, USA) were made by dispersing agarose powder in PBS and microwaving at 350 W power in 5- to 8-s intervals until dissolved. Stocks were stored at 4°C and could be re-melted at 95°C using the same microwaving method. The molten agarose remained liquid down to 35°C. Gels of 1%, 0.5%, and 0.2% concentration were formed by melting stock gels and mixing with warm PBS (35°C) directly on glass slides for imaging, kept warm on a hotplate set to 35°C, or directly on a heated rheometer stage in the case of rheology measurements. Molten gels were taken off heating apparatus to cool to ambient temperature to induce gelation. Gel osmolality, which influences degree of vesicle deflation, was varied with the addition of glucose as opposed to salts to maintain the ionic strength of the solution, avoiding electrostatic screening effects. Solution osmolality before the addition of agarose was adjusted with a freezing-point osmometer (Osmomat 3000, Gonotec, Berlin, Germany). A list of tested gel formulations can be found in Table 2 in the Supporting Material (section S5).

Bulk rheology of agarose hydrogels was studied in shear mode using an Anton Paar MCR301 rheometer with 12-mm cone-plate (CP12) geometry (Anton Paar, Graz, Austria). Gels were mixed directly on the rheometer stage heated to 35°C, then cooled below 20°C to allow the sample to start to set while the probe was lowered to the measurement position on the sample. The gel was left for 5 min to fully set before testing up to 1% rotational strain from 1 to 10 Hz.

Average gel pore size was estimated using a turbidimetric assay described by Aymard et al. (29) and Narayanan et al. (27) Briefly, molten agarose was added to disposable 2.5-mL PMMA cuvettes (Sigma-Aldrich, St. Louis, MO, USA) and allowed to cool to ambient temperature (∼22°C) to gel. Absorbance values over 600–900 nm were measured using a Thermo Spectronic Helios Gamma UV-Vis Spectrophotometer (Thermo Fisher Scientific, Waltham, MA, USA). This was compared to analytical data from Aymard et al. (29) (see section S2, Supporting Material).

### Quantifying particle mobility

LUVs were embedded in agarose gels by mixing extruded LUV solutions with molten agarose directly on a glass microscopy slide within a rubber spacer (see section S3, Supporting Material). A glass coverslip was placed on top, such that the agarose droplet wetted both glass surfaces, forming a disk. The imaging chamber was set aside at room temperature for 5 min to set. For control experiments with embedded polystyrene beads, working mixtures of Fluoresbrite YG 0.1-*μ*m-diameter Microbeads (Polysciences, Warrington, PA, USA) and FluoSpheres carboxylate-modified 0.1-*μ*m-diameter red (580/605) polystyrene beads (Invitrogen, Waltham, MA, USA) were made by diluting bead suspensions 1:100 in PBS before being mixed into gels, replacing the LUV solution at the same volume.

Samples were imaged with a pco.edge sCMOS camera mounted to a Zeiss AXIO Observer.D1 microscope with a 63× water immersion objective (Carl Zeiss, Oberkochen, Germany) in epifluorescence mode with appropriate excitation and emission filters. Image sequences of length 5 s (∼100 frames) were captured with 20-fps frame rate and ∼45-ms exposure in a 100 × 100-*μ*m region of interest (ROI). Three ROIs were recorded per sample to account for internal heterogeneity. Particle mobility within gels was analyzed with the single-particle tracking plugin for FIJI developed by Sbalzarini and Koumoutsakos (25). A sequence length of 100 frames was chosen because longer sequences resulted in decreased signal-to-noise ratio from photobleaching, leading to increased false positives in particle detection. Histograms of the log_10_ diffusion coefficients (in m^2^ s^−1^) obtained from the plugin were used to determine the mobile fraction.

Due to their size being below the diffraction limit, fluorescently labeled particles with a nominal diameter of 100 nm appear in images with pixel size 100 × 100 nm as small clusters of 3–4 pixels with approximately 1- to 2-pixel spread (see section S3, Supporting Material). In order for motion to be detected above the noise floor, a particle must be displaced more than 3 pixels from its original position, occurring over *t* = 2.5 s. This corresponds to the maximum lag time used in the calculation of the mean squared displacement (MSD), or half the total duration of the image sequences used. A theoretical lower limit of detection of particle movement can thus be calculated using the following relationship between the MSD and the diffusion coefficient, *D*, in two dimensions (43):

$$D_{\min}=\frac{{MSD}_{\min}}{4t}=\frac{{\left(\min.\;displacement\right)}^{2}}{4t}=\frac{{\left(300nm\right)}^{2}}{4\left(2.5s\right)}=9.0\times 10^{-15}m^{2}s^{-1}$$

We thus use $\log_{10}\left(D_{\min}\right)\approx -14$ as the cutoff point to determine whether a particle is mobile.

We also studied the infiltration of 100-nm-extruded DOPC LUVs into preformed 1% w/v agarose hydrogels to obtain a collective diffusion coefficient. The prior imaging chamber setup was done with slight adjustments (see Fig. S4). Briefly, agarose gel disks were formed without LUVs in an imaging chamber and allowed to set before a solution of LUVs (12 *μ*M lipid) was pipetted into the chamber. Three 100 × 100-*μ*m ROIs each in the gel interior and the exterior solution were imaged per time point, per sample over 100 h. The number of particles in each ROI inside and outside the gel was counted as a function of time. Because of the slow diffusion and the low number of particles reaching the gel interior, we were unable to satisfactorily discretize the images to obtain smooth particle density gradients, as previously done for fluorescently labeled molecules (44). Instead, we obtained density gradients using finite differences and computed the diffusion coefficient as follows: each ROI is a rectangular box with dimension h=100μm and L=100μm. Assuming the depth of the observation volume is held constant, the system can be reduced to a two-dimensional model, whereby the two-dimensional flux per unit area, *J*, flowing into the ROI in the gel interior is given by

$$J=\frac{1}{L}\frac{dN_{in}}{dt}$$

where $N_{in}$ is the number of LUVs in the gel interior and *t* is time. Due to the slow diffusion, $N_{in}$ appears to vary linearly in time (see Fig. S4 *D*), hence

$$\frac{dN_{in}}{dt}=\frac{N_{in}}{t}$$

Fick’s first law of diffusion in two dimensions connects the density $\varphi =\frac{N}{hL}$ with the flux by introducing a diffusion constant, D, via

$$J=-D\frac{d\varphi }{dx}=-\frac{D}{hL}\frac{dN}{dx}$$

Finally, we approximate the density gradient using a finite difference

$$\frac{dN}{dx}=\frac{N_{in}-N_{out}}{x}$$

where x=300μm is the distance between the ROI of the gel interior and the edge of the gel and $N_{out}$ is the number of particles in the ROIs in the exterior LUV solution. Combining these relations and solving for the diffusion constant gives

$$D=-\frac{N_{in}hx}{\left(N_{in}-N_{out}\right)t}$$

Because of the slow diffusion, all initial time points where $N_{in}=0$ are excluded. Diffusion coefficients were calculated at each time point measured (4–13 data points per replicate over 100 h of imaging; see Fig. S4 *D* in Supporting Material), then averaged for each replicate.

### Statistical analysis

Histograms of log_10_ diffusion coefficients are normalized to show probability and represent pooled data from three ROIs within an individual gel. Each ROI corresponds to 100–300 diffusing particles. The variability in the number of identified particle tracks in different gels arises from differences in particle mobility. Statistics on mobile fractions were calculated with *n* = 3 gels, presented as standard boxplots showing the median (middle line), upper and lower quartiles (box limits), and full range of non-outlier data (whiskers). All other data are presented as mean with standard deviation. Statistical significance was determined with N-way ANOVA (as indicated) with Tukey-Kramer tests for multiple comparisons at the significance levels indicated, computed using MATLAB (MathWorks, Natick, MA, USA).

## Results and discussion

### Mobility of embedded LUVs

**Figure 1:**
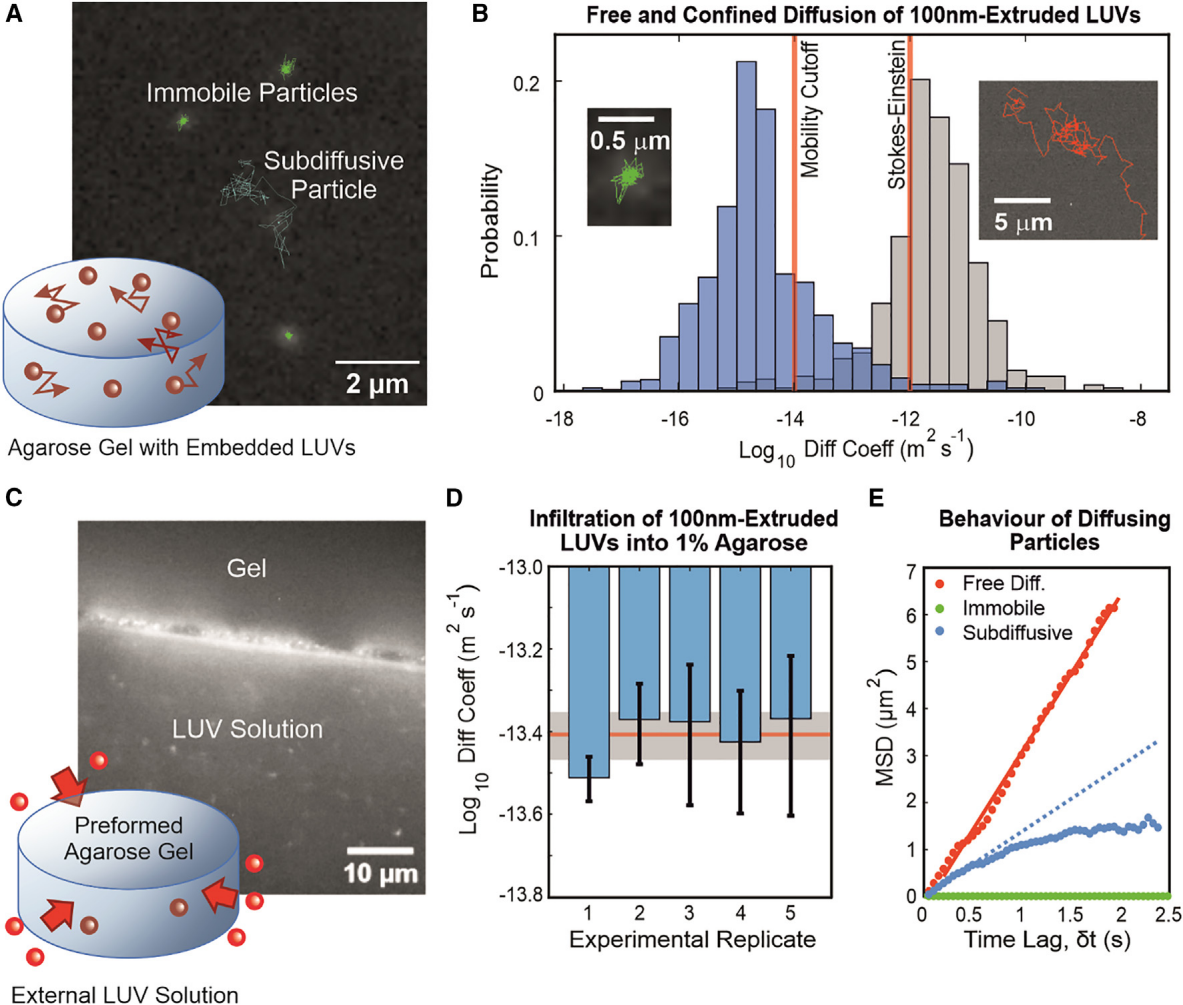
Particle mobility assessed in two ways: diffusion of DOPC LUVs embedded in gels and infiltration of LUVs into gels. (*A*) The diffusion of 100-nm extruded DOPC LUVs embedded in 1% agarose gels is analyzed with single-particle tracking. Particle paths are indicated as an overlay on a representative time frame. Here, green trajectories represent immobilized LUVs and a subdiffusive trajectory is shown in blue. This particular LUV was observed to “jump” between two hydrogel-pore regions, resulting in this biphasic trajectory separated by a particularly large displacement. (*B*) The base-10 logarithms of the diffusion coefficients are shown in histogram form for 100-nm extruded LUVs embedded in 1% agarose (*blue*) and in agarose-free liquid PBS (*gray*). The values of −14 and −12 are indicated by red vertical lines, the former being the lower limit of detection of particle motion for the experimental setup and the latter being the theoretical value determined from the Stokes-Einstein equation for an ideal 100-nm-diameter spherical particle diffusing in liquid water. The insets show fragments of two trajectories corresponding to an immobilized/confined LUV in 1% agarose (*green*, *left*) and a freely diffusing one in agarose-free PBS (*red*, *right*). (*C*) A measure of the collective particle diffusion is determined by incubating preformed agarose gel disks with an external solution of LUVs and monitoring their infiltration into the gel. The epifluorescence microscopy image shows the edge of an agarose gel disk, where LUVs can be seen adsorbed onto the surface. Individual LUVs in the solution are seen as tiny spots. (*D*) The particle densities inside and outside the gel over time can be related to the diffusion coefficient (see section S2). The average log_10_ diffusion coefficients computed from five independent experimental replicates are presented here with error bars showing standard deviation. The ensemble average across all experimental replicates was found to be 3.92 ± 0.52 × 10^−14^ m^2^ s^−1^ (indicated as a red line with gray shaded area showing standard deviation), corresponding to a log_10_ value of −13.41. (*E*) In both experiments, LUVs can be observed to exhibit different diffusive behaviors, characterized by different MSD plots. A freely diffusing LUV is characterized by a linear MSD plot, as shown in red, where the slope is proportional to the diffusion coefficient. The green plot shows a fully immobilized particle in agarose, where the slope is very close to zero. The blue MSD plot shows subdiffusive or anomalous diffusion behavior, whereby the MSD scales with a power of time, $MSD\propto {\delta t}^{r}$, where r < 1. A blue dotted line shows a linear fit of the first 10 data points of the MSD plot to illustrate the difference between subdiffusion and regular diffusion. The effective diffusion coefficient would be calculated and fitted over the whole MSD curve, resulting in an overall lower diffusion coefficient than would be expected of a freely diffusing particle. To see this figure in color, go online.

LUVs embedded inside agarose gels were imaged with epifluorescence microscopy and analyzed with single-particle tracking (25) to obtain their diffusion coefficients (Fig. 1 *A* and *B*). The mobile fraction was determined from the distribution of log_10_ diffusion coefficients. The peak observed at a value of −15 for 100-nm-extruded LUVs composed of pure DOPC embedded in 1% agarose (Fig. 1 *B*) lies below the mobility cutoff of −14 (see Supporting Material, section S1) and thus corresponds to fully immobilized particles. Particle immobilization and interactions in general with a gel matrix have previously been described in terms of electrostatic effects (at least for polystyrene particles) (45). In essence, although some particles can become fully entrapped by the gel matrix, other particles will be able to diffuse unhindered within the matrix voids as if they were in liquid water. Given close enough proximity to a wall or surface, particles can transiently bind and unbind with the gel matrix, reducing their MSD and thus “effective” diffusion coefficient. It is possible in our case with flexible lipid vesicles that these interactions can also be steric, with transient trapping and freeing of particles due to thermal fluctuations. The obtained effective diffusion coefficient can thus be used as a measure of the frequency and strength of membrane-matrix interactions, including steric ones. Since virtually all particles lie below −12 (Fig. 1 *B*), the value given by the Stokes-Einstein equation for an ideal 100-nm spherical particle diffusing in liquid water, this implies that all particles in the system are interacting with the gel matrix, sterically or otherwise.

**Figure 2:**
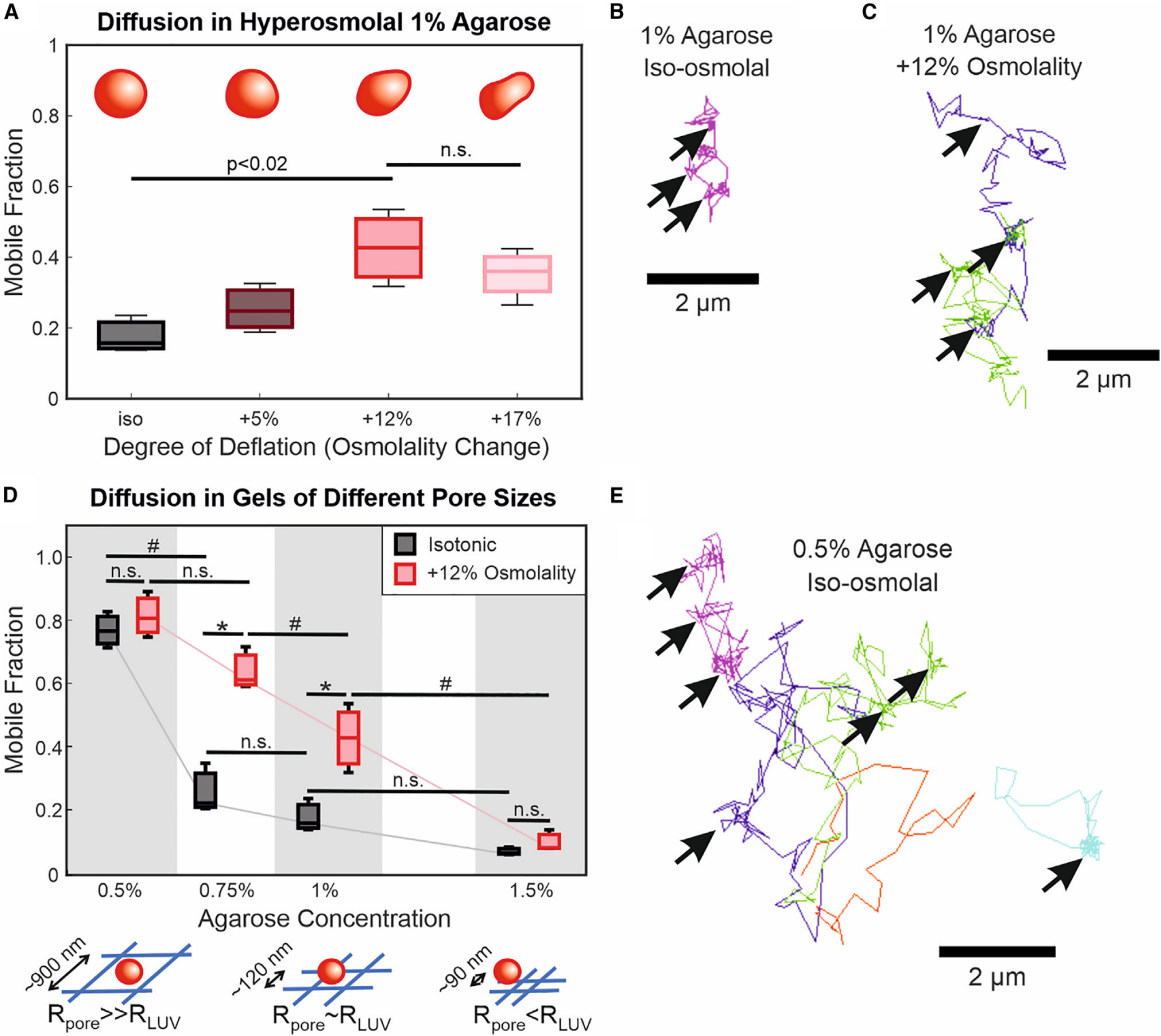
Mobility of DOPC LUVs increases with osmotic deflation and with hydrogel pore size. (*A*) Mobile fractions of LUVs in agarose gels with different osmolalities relative to the LUV interior with cartoon representations of an LUV at varying stages of deflation. (*B*) Example trace of a particle trajectory in 1% iso-osmolal agarose with arrows indicating apparent particle trapping regions. This trajectory may not be representative of the majority of particles in this condition, as it is specifically a particle with relatively high mobility to illustrate trapping behavior; hence, the apparent pore size traced by the particle trajectory may not correspond to the average size determined with turbidimetry. (*C*) Representative particle trajectories in 1% agarose with 12% osmolality increase compared to the intravesicular solution. Different colors represent different particles and arrows indicate apparent trapping points. (*D*) Mobile fractions of LUVs in agarose gels of differing concentration and osmolal strength with cartoon representations comparing the relative sizes of pores and LUVs. Statistically significant difference between osmolalities at constant agarose concentration, (p < 0.01) is indicated with asterisks (∗); statistically significant difference between agarose concentrations at constant osmolality, (p < 0.05) is indicated with the pound sign (#), as determined with one-way ANOVA and pairwise Tukey-Kramer post hoc analysis. Lack of statistically significant difference (p > 0.05) is indicated with n.s. (*E*) Representative particle trajectories in 0.5% iso-osmolal agarose with arrows indicating apparent trapping points. Different colors represent separate particles. To see this figure in color, go online.

Analyzing individual particle trajectories reveals different diffusive behaviors. Freely diffusing particles in liquid can be observed covering large areas, whereas immobilized particles in gels remain stationary. Most LUVs embedded in gels undergo anomalous diffusion or subdiffusive behavior, whereby particles diffuse within the confines of a matrix pore, resulting in a characteristic MSD curve with a plateau at long time lags (Fig. 1 *E*). These particles can sometimes “hop” between pore spaces, similar to what has been described in polystyrene nanoparticles diffusing in liquid polymer solutions (12) and in hydrogel matrices (14). Examples can be seen in Figs. 1 and 2. We note that particles appear to also become transiently trapped at certain locations within presumed pore spaces. These particles dwell at these locations for multiple consecutive image frames for periods of 0.25 s, up to several seconds long, sometimes alternating between several trapping points before being freed. This appears to occur at size scales smaller than the apparent pore sizes mapped out by the rest of the particle’s trajectory or by neighboring particles.

### Collective LUV diffusion from gel infiltration

We next looked at the ability of DOPC LUVs to infiltrate preformed agarose hydrogel disks to obtain an independent measure of particle mobility based on population dynamics (Fig. 1 *C* and section S2). Although many LUVs ended up adhering to and getting stuck at the edge of the agarose disks, some LUVs were observed to infiltrate into them over 100 h of imaging. Equilibrium in the density gradient was not reached in the time frame tested. By approximating the LUV density gradient with a finite difference, we calculate a population-wide diffusion coefficient using Fick’s first law of diffusion to be 3.92 ± 0.52×10^−14^ m^2^ s^−1^ (see Fig. 1 *D* and section S4), corresponding to a log_10_ value of −13.41. This falls between the mobility cutoff and the value for the Stokes-Einstein particle, agreeing well with the results from the single-particle tracking of embedded LUVs (Fig. 1 *A* and *B*).

### Osmotic deflation of LUVs increases diffusivity

**Figure 3:**
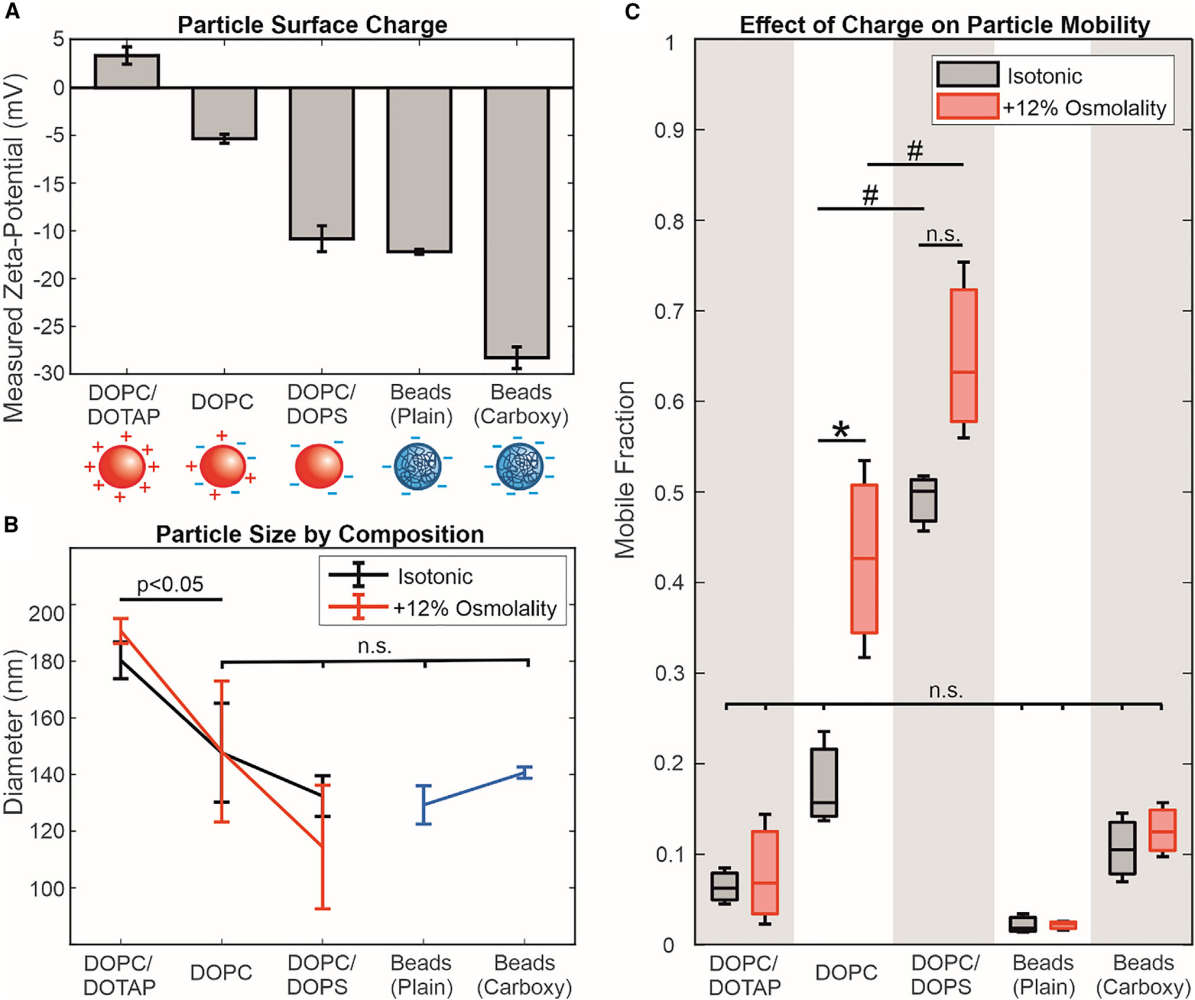
Effect of charge and composition on particle mobility in 1% agarose gels. The lipid ratios in DOPC/DOTAP and DOPC/DOPS LUVs correspond to 2:1. (*A*) Zeta potential of LUVs and polystyrene beads (either plain or with surface carboxylation), as measured in PBS; the sketches below roughly illustrate their surface charge. (*B*) Diameters of LUVs and particles, as measured with DLS. Average diameters of DOPC/DOPS and DOPC LUVs, as well as the polystyrene beads (*blue*), are not significantly different, in both isotonic (*black*) and hypertonic (*red*) conditions, as determined by two-way ANOVA with pairwise Tukey-Kramer post hoc analysis (p > 0.05). Representative size distributions of particles can be found in Fig. S6 in the Supporting Material. Sizes of DOPC/DOTAP LUVs are significantly larger than those of the other particles (p < 0.05). (*C*) Mobile fractions of different particles in isotonic (*black*; 290 mOsm/kg) and +12% hypertonic (*red*; 320 mOsm/kg) buffer conditions; see Fig. S5 for diffusivity data. Statistical significance was determined with two-way ANOVA with pairwise Tukey-Kramer post hoc analysis. Significant difference across membrane compositions, (p < 0.01) are denoted with the pound sign (#). Astatistically significant difference between different osmolalities, (p < 0.02) is denoted with asterisks (∗); lack of a significant difference (p > 0.02) is denoted with n.s. The lack of a statistically significant difference between DOPC/DOPS LUVs in isotonic and hypertonic environments was confirmed with a paired-sample *t*-test, which did not reject the null hypothesis (p > 0.05). Note that, although the maximum extents of the data (*whiskers*) do not overlap, the range of the 95% confidence intervals (not shown for clarity) do overlap. To see this figure in color, go online.

Work by Yu et al. (9) on LUV compositions of different phase transition temperature and by Lenzini et al. (10) on cell-derived EVs has shown that vesicle deformability can affect their diffusion in a hydrogel. Another way to make LUVs more deformable is to deflate them by introducing them into a hypertonic environment. We studied the mobility of DOPC LUVs in agarose gels of differing osmolalities by adding glucose to the hydrogel solution while keeping the initial intravesicular solution constant. Fig. 2 *A*–*C* shows that LUV mobility increases with osmolality from isotonic to +12% osmolality and thus degree of deflation. No significant difference in LUV size was detected with DLS, although size distributions appear to have slightly higher variability in hypertonic solutions (Fig. 3 *B* in the main text; size distributions found in Fig. S5 *C* in the Supporting Material). We also did not observe differences in the bulk rheology of agarose gels formed with and without glucose (Fig. S5 *A*); thus, the microstructure of the gel is not expected to vary more than what is naturally found in agarose (26). The lack of statistically significant difference in mobility from +12% to +17% osmolality (Fig. 2 *A*) could be due to a phenomenon similar to what was described by Yu et al. (9), whereby greater deformability ultimately exposes greater surface area that can conform to and interact with the matrix walls, resulting in immobilization. Recent theoretical work (18) also shows that particle diffusibility in a gel matrix is dependent on a balance of particle deformation and adhesive forces in the matrix. Kinetic energy in a hyper-deformable vesicle’s collision with a matrix wall could end up being spent on deforming the membrane, such that insufficient energy remains for overcoming matrix-adhesive forces.

When the pore size of the gel is increased from 120 ± 6 nm to 250 ± 30 nm by decreasing the agarose concentration from 1% to 0.75% w/v (Fig. S2), the mobility of LUVs does not increase significantly in isotonic conditions but does so under hyperosmotic conditions (Fig. 2 *D*). A further increase in pore size to 900 ± 300 nm by decreasing the agarose concentration to 0.5% results in an overall increase in LUV mobility. At this concentration of agarose, the difference between the mobile fractions in different osmolalities is not statistically significant. The effect of deflation thus appears to only be relevant when the average pore diameter is comparable (i.e., on the same order of magnitude) to the diameter of the LUV. This is reasonable, as the LUVs would have greater access to matrix pores regardless of deformability in the 0.5% gel (Fig. 2 *E*). At the opposite extreme, decreasing the average pore size to below the average LUV diameter to 90 ± 6 nm results in nearly full immobilization of LUVs, even when osmotically deflated.

### LUV surface charge affects mobility

**Figure 4:**
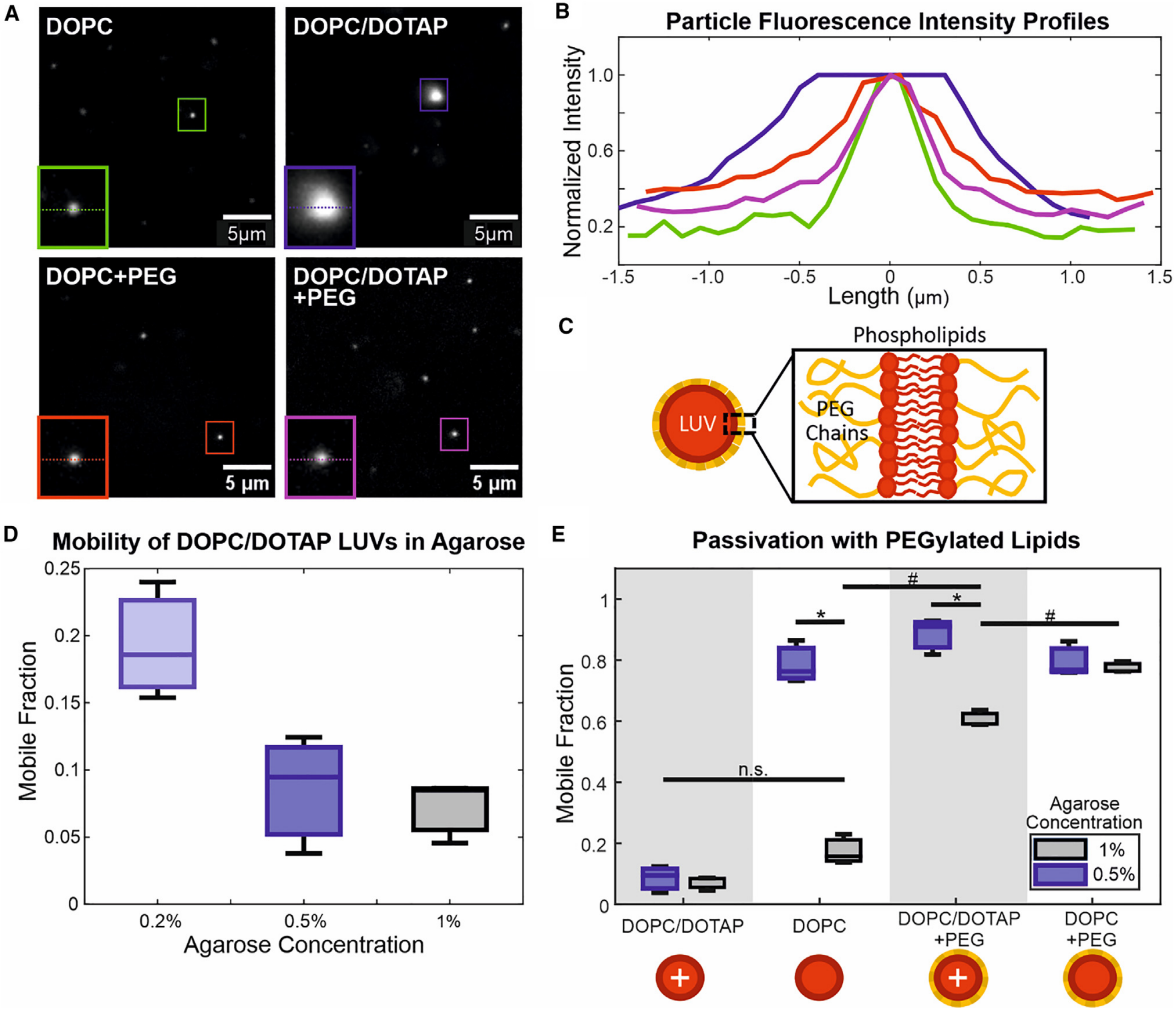
Recovery of DOPC/DOTAP LUV mobility with PEGylated lipids. (*A*) Representative images of 100-nm-extruded DOPC, 2:1 DOPC/DOTAP, DOPC + 10 mol % DSPE-mPEG1K (DOPC + PEG), and 2:1 DOPC/DOTAP + 10 mol % DSPE-mPEG1K (DOPC/DOTAP + PEG) LUVs in 0.5% agarose gels. Selected particles are indicated with colored boxes and shown in insets (2.9-*μ*m width), where a dotted line indicates the position at which the fluorescence intensity profiles in (*B*) are taken. Particles appearing with different absolute intensities could be due to being at different focal depths or because of different degrees of photobleaching, as particles move in and out of frame or focus. The selected DOPC/DOTAP particle is likely an aggregate of LUVs. (*B*) Normalized fluorescence intensity profiles of selected LUVs. Colors correspond to the particles in (*A*). The DOPC, DOPC + PEG, and DOPC/DOTAP + PEG profiles represent single particles, which are smaller than the diffraction limit of our imaging setup. These particles thus appear blurry with considerable spread but with comparable normalized fluorescence intensity profiles. The DOPC/DOTAP aggregate, by contrast, has a diameter approaching 1 *μ*m and is noticeably larger by visual inspection. (*C*) Schematic diagram of a LUV membrane containing PEGylated phospholipids. (*D*) Mobile fractions of 2:1 DOPC/DOTAP LUVs in agarose gels/solutions of differing concentration. Agarose at 0.2% concentration behaves macroscopically as a liquid but likely has some weak molecular network linkages. (*E*) Comparison of mobile fractions of 2:1 DOPC/DOTAP LUVs (DOPC/DOTAP), pure DOPC LUVs (DOPC), 2:1 DOPC/DOTAP LUVs + 10 mol % DSPE-mPEG1K (DOPC/DOTAP + PEG), and DOPC LUVs + 10 mol % DSPE-mPEG1K (DOPC + PEG). Hash (#) indicates statistically significant differences when comparing mobilities across LUV compositions in the same gel concentration. Asterisk (^∗^) indicates statistically significant differences when comparing mobilities of LUVs in different agarose gel concentrations. Mobility of DOPC/DOTAP LUVs in both 1% and 0.5% agarose, as well as that of DOPC LUVs in 1% agarose, are not significantly different (n.s.). Statistical significance is determined with two-way ANOVA with pairwise Tukey-Kramer post hoc analysis for multiple comparisons. To see this figure in color, go online.

Lieleg et al. (8) reported that Matrigel, a complex mixture of cell-derived ECM materials, exhibits electrostatic filtering behavior on diffusing particles. This has also been shown with polymer solutions and in computer simulations (11). To determine whether agarose has similar characteristics, we produced negatively charged LUVs from a 2:1 molar ratio mixture of DOPC/DOPS as well as positively charged LUVs composed of 2:1 DOPC/DOTAP to embed in agarose (Fig. 3 *A*). The addition of DOPS does not significantly change LUV size (Fig. 3 *B*), but the DOPC/DOTAP particles appear significantly larger than other tested particles, possibly due to aggregation. Fig. 3 *C* shows that the negatively charged DOPC/DOPS LUVs have greater mobility overall compared to the positively charged DOPC/DOTAP and pure zwitterionic DOPC LUVs (see also Fig. S6). Although there appears to be an increase in mobility in DOPC/DOPS LUVs upon deflation, this difference is not statistically significant according to a one-way ANOVA test with pairwise comparisons over the whole dataset (p > 0.05). This was also confirmed with a paired-sample *t*-test with just the DOPC/DOPS data, which did not reject the null hypothesis (p > 0.05). DOPC/DOTAP LUVs remain immobile in hypertonic conditions as well as in much lower agarose concentrations (Fig. 4 *C*). At 0.5% w/v, agarose would have an average pore size of 900 nm, which should be large enough to accommodate even the largest aggregates detected with DLS (∼300 nm, see size distributions in Fig. S6 *B*). At 0.2% w/v, agarose behaves like a liquid, being able to flow. The lack of mobility at these concentrations would suggest that this interaction is not merely steric but electrostatic, causing the positively charged LUVs to stick to the agarose polymer bundles. This could help explain the apparent size of DOPC/DOTAP LUV aggregates seen in agarose gels (Fig. 4 *A*) but not in suspension. Analysis of fluorescence intensity profiles of particles embedded in agarose shows that such aggregates approach 1 *μ*m in diameter, whereas the maximum particle size detected with DLS is approximately 300 nm.

As a control, we compared LUV mobility to that of polystyrene beads. Despite having a similar size and negative surface charge to our DOPC/DOPS LUVs, plain polystyrene beads are fully immobilized in the hydrogel compared to the highly mobile DOPC/DOPS LUVs. Beads with surface carboxylation appear to have slightly higher mobility than plain beads but not at a statistically significant level. Neither types of beads are affected by increased osmolarity. One explanation for this relates to the fact that the polystyrene beads are rigid but the LUVs are deformable and capable of squeezing through gel matrix pores that would otherwise be too small to pass through. The increased surface charge of the DOPC/DOPS compared to DOPC LUVs should also result in a slightly stiffer membrane (46), although this effect could be minimized by the high salt concentration. In 0.5% agarose gels, plain polystyrene beads remain immobile (mobile fraction = 0.07 ± 0.05) despite the much larger pore size, whereas carboxylated beads become much more mobile (mobile fraction = 0.87 ± 0.08; see Fig. S6 in Supporting Material). It is possible that the enhanced negative charge of the carboxylated beads overcomes specific attractive interactions present between the agarose gel and the polystyrene beads. Another possibility relates back to the theoretical work of Yu et al. (18), who showed that highly rigid particles require very low attractive forces to maintain diffusibility. These results reveal an interesting intersection of different factors affecting the diffusion of particles through a gel matrix. In particular, we note that the use of polystyrene nanoparticles to model the diffusion of LUVs, EVs, and other soft particles may not be accurate due to the differences in their overall deformability, even if their surface properties are matched.

### PEGylation of LUVs increases their mobility

Nanoparticles are often passivated with PEG (polyethylene glycol), a hydrophilic polymer used to prevent the adsorption of proteins on surfaces and hinder or slow down immune reactivity. It has been claimed that this passivation effect is due to the neutral charge of PEG masking the underlying surface (8,19,22), although other works suggest the involvement of steric or entropic effects of the PEG chains (13,47). We questioned whether this effect could restore the mobility of our DOPC/DOTAP LUVs (Fig. 4 *C*). For this to work, the layer of PEG chains would need to be thicker than the Debye length of the charges on the membrane. The inclusion (1,2-distearoyl-sn-glycero-3-phosphoethanolamine-N-[methoxy(polyethylene glycol)-1000]) (DSPE-mPEG1K), a phospholipid coupled to a 1000-Da PEG chain at 10 mol %, should make a PEG layer thick enough to screen out most electrostatic effects. In the high ionic strength buffer environment (PBS) that was tested, the Debye length, the distance over which an electric charge exerts an influence, is <1 nm (48). Meanwhile, the three-dimensional Flory radius of the polymer, *R*_*F*_ is given by $R_{F}\approx a_{m}n_{p}^{3/5}$, where *a*_*m*_ is the size of the monomer unit (*a*_*m*_ ≈ 0.39 nm for PEG, as used by Marsh et al. (49)) and *n*_*p*_ is the number of monomers in the polymer (∼23 for PEG1000). The mean-field theory equilibrium length of the polymer chain, *L*^*MF*^, describing the average height of the polymer brush layer, is also given by Marsh et al. as $L^{MF}\approx n_{p}a_{m}^{5/3}{\left(X_{p}/A_{l}\right)}^{1/3}$, where *X*_*p*_ is the molar fraction of PEGylated lipid and *A*_*l*_ is the area per lipid molecule of the membrane, taken to be ∼0.6 nm^2^ for a lipid in the fluid phase. Both *R*_*F*_ and *L*^*MF*^ are approximately 2.6 nm for PEG1000 at 10 mol % coverage in a fluid-phase lipid membrane, and thus greater than the Debye length. Therefore, the PEG layer should be thick enough to block electrostatic interactions with the underlying phospholipid surface. This indeed appears to restore the mobility of the DOPC/DOTAP LUVs in 0.5% w/v agarose to the same level as pure DOPC LUVs (and DOPC + PEG LUVs; Fig. 4 *D*). The mobility of the passivated DOPC/DOTAP + PEG LUVs in 1% agarose is much improved compared to that of the nonpassivated DOPC and DOPC/DOTAP LUVs, but it is lower than the passivated DOPC + PEG LUVs. One possible explanation for this is that LUVs need to deform to fit through the smaller pores of the 1% agarose. This could force the PEG layer to compress or deform out of the way, exposing the underlying positive charge. This is possible, as previous work has shown that PEG is highly compliant (20,23). Furthermore, the addition of PEG appears to prevent the particle aggregation apparent in DOPC/DOTAP LUVs, bringing the mean peak value of the size distribution closer to the expected value of 100 nm (Fig. S6; Supporting Material). It also seems to prevent aggregation of particles upon embedding in agarose (Fig. 4 *A* and *B*). Since free particles have a diameter below the diffraction limit of our imaging setup, we cannot directly compare their apparent sizes in images. Direct comparison of fluorescence intensity is also not possible due to the particles appearing at different focal depths with our epifluorescence imaging and because of photobleaching. However, DOPC/DOTAP LUV aggregates are apparent upon visual inspection and analysis of their fluorescence intensity profiles shows that they have diameters approaching 1 *μ*m. Aggregates of these sizes are not detected by DLS and are not apparent with visual inspection of samples in liquid suspension, only appearing upon embedding in agarose. These aggregates, thus, are likely the result of electrostatic interactions with the agarose matrix, causing the pinning together of particles. This effect disappears upon addition of PEGylated lipids, as the PEG prevents both particle-particle and particle-matrix interactions.

**Figure 5:**
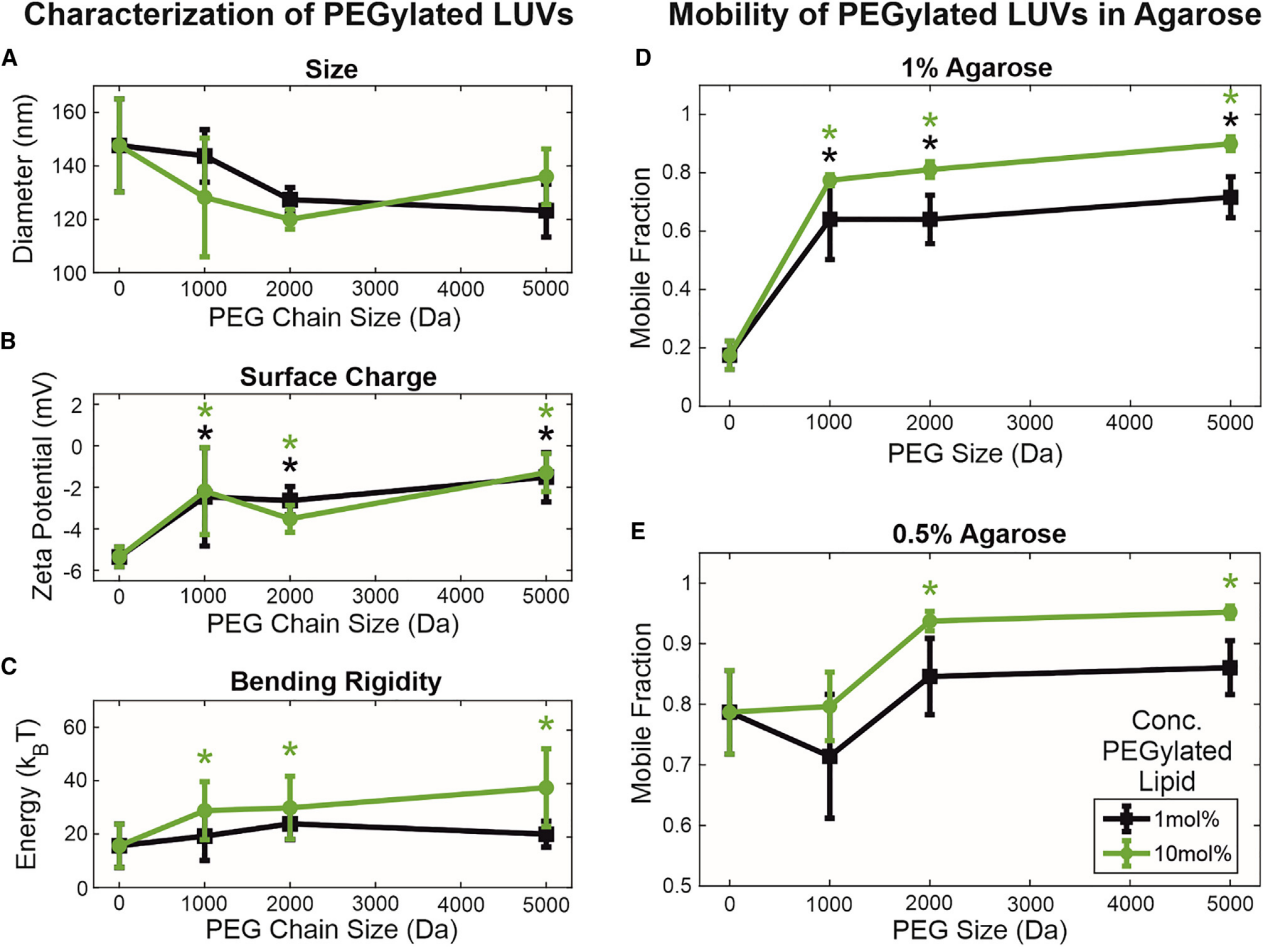
Effect of degree and density of PEGylation on LUV characteristics and mobility. Green data represent 10 mol % concentration of PEGylated lipid in membranes, whereas black data represent 1 mol % concentration. (*A*) Influence of PEG chain size and concentration on LUV size, as measured with DLS. No statistically significant differences were found (p > 0.05) and size distributions appear similar (Fig. S7*B* in Supporting Material). (*B*) Influence of PEG chain size and concentration on surface charge, measured as the zeta potential. Bare DOPC LUVs are significantly more electronegative compared to PEGylated LUVs, regardless of PEG size and concentration (p < 0.01, *green and black asterisks*), demonstrating screening by PEG. (*C*) Influence of PEG chain size and concentration on membrane bending rigidity, measured with fluctuation analysis on GUVs and presented in k_B_T energy units (k_B_ being the Boltzmann constant and T being temperature). Membranes with 10 mol % PEGylated lipid are significantly stiffer than those with 1 mol % PEGylated lipid. Bare DOPC membranes are significantly less stiff than PEGylated membranes only at 10 mol % concentration (p < 0.01). No statistically significant differences were found between different PEG chain sizes. Statistically significant differences compared to bare DOPC membranes were determined with two-way ANOVA with pairwise Tukey-Kramer post hoc analysis, as indicated with asterisks (^∗^). (*D*) Mobilities of all PEGylated particles in 1% agarose are significantly greater than those of bare DOPC LUVs at both concentrations of PEGylated lipid (p < 0.01, *green and black asterisks (^∗^)*). Mobilities of LUVs with 10 mol % PEGylated lipid are significantly greater than those of LUVs with 1 mol % PEGylated lipid (p < 0.01). (*E*) Mobilities of LUVs with 10 mol % PEG2000 and PEG5000 are significantly greater than those of bare LUVs and LUVs with 10 mol % PEG1000 (p < 0.01). No significant differences were found at 1 mol % PEGylated lipid. Statistically significant differences compared to bare DOPC membranes are indicated with asterisks (^∗^), as determined by three-way ANOVA with pairwise Tukey-Kramer post hoc analysis. To see this figure in color, go online.

Although PEGylation improved the mobility of DOPC/DOTAP LUVs, it also improved that of DOPC LUVs despite previous results of higher mobility in agarose with greater negative charge. DOPC LUVs also do not appear to aggregate as DOPC/DOTAP LUVs do, so this effect cannot be due to the prevention of particle aggregation. To investigate further how PEG affects LUV mobility, we tested other PEG chain sizes (1000, 2000, 5000 Da) at two concentrations (1 mol % and 10 mol %; Fig. 5 *A*–*C*). Fig. 5 *D* and *E* shows that all PEG chain sizes improve LUV mobility in 1% agarose, whereas LUVs with PEG2000 and PEG5000 appear to be more mobile than those with PEG1000 in 0.5% agarose gels (see histograms and size distributions in Fig. S7). LUV mobility is higher with 10 mol % PEGylated lipid, where the PEG chains are in a polymer brush conformation (20,23,49) compared to 1 mol % polymer, where they are in mushroom conformation (20,23,49). The difference in PEG chain conformations could help explain why PEG1000 appears ineffective in 0.5% agarose, as the mushroom-to-brush transition occurs at a higher concentration and is less well defined. Regarding the distributions of the log_10_ diffusion coefficients (Fig. S7 *A*), although a greater proportion of particles become mobile upon addition of PEGylated lipids, the mean peak value of the mobile particles does not appear to change for a given concentration of agarose. For LUVs in 1% agarose, the peaks of the distributions fall consistently below −12 and appear to shift toward greater mobility, approaching −12 when the agarose concentration is decreased to 0.5%. This does not appear to be affected by size or surface coverage of PEG chain. Although the PEG might prevent matrix interaction and immobilization, the agarose gel would continue to impose steric hindrance and restrict the space a particle can diffuse in, resulting in subdiffusion and, thus, a lower effective diffusion coefficient. When this steric hindrance is lessened by the much larger matrix pores of 0.5% agarose, particles are able to diffuse unrestricted over a much larger area, approaching the Stokes-Einstein-predicted value of −12 for a similarly sized particle in liquid.

These results suggest that the effect that PEG has on particle mobility is not entirely electrostatic. Greater mobility is also unlikely to be due to disaggregation of particles, since DOPC LUVs do not appear to aggregate without PEG upon visual inspection and according to the lack of change in average size and size distributions measured with DLS (Figs. 5 *A* and S7 *B*).

Fluctuation analysis on GUVs composed of the same lipid mixtures as the tested LUVs shows a slight stiffening of lipid membranes with the presence of PEGylated lipids (Fig. 5 *C*). This behavior is consistent with predictions for membrane stiffening by anchored polymers (50) and experiments on membranes with biopolymer adsorption (51) as well as microemulsions (52). However, the stiffening observed here does not explain the increase in LUV mobility measured when PEGylated lipids are present. As demonstrated in Fig. 2, mobility should improve with overall deformability, which would decrease with increasing bending rigidity. One possibility would be that the PEG forms a soft lubricating layer that facilitates the movement of LUVs through matrix pores. This would be supported by the higher mobile fraction observed with larger PEG chains and at higher PEG coverage (in the brush regime), which would form a thicker layer. The underlying mechanism for this could be explained by the entropic repulsive force described in the computational work of Li and Shi (47), whereby the compression of the PEG layer during a collision with the matrix wall would produce a strong repulsive force, preventing entrapment of the LUV in the matrix. It has also previously been reported that surface PEGylation improves the mobility of nanoparticles in mucin gels by sterically preventing the adsorption of colloidal mucin onto the particle surface (13). These results, although similar, are likely due to a different mechanism, as agarose is not known to exist in a colloidal phase capable of adsorbing onto the particle surface and should be fully incorporated into the matrix scaffold (26,30). A particularly interesting question for future consideration would be whether the glycocalyx, the diverse array of polymeric sugar molecules expressed on the surfaces of cells and cell-derived EVs, might play analogous roles in vivo.

## Conclusions

Here, we have demonstrated the applicability of agarose as a bio-inert and nonadhesive model for investigating the nonspecific steric and electrostatic interactions of vesicles with a 3D polymer matrix. The use of a bottom-up biomimetic approach to study lipid vesicle diffusion in hydrogel materials has shown that different biophysical factors contribute to particle dynamics. Even without specific biochemical interactions via cell adhesion molecules and ECM proteins, the combined effects of nonspecific steric and electrostatic interactions can give rise to selective filtering behavior, allowing particles of certain surface charge characteristics and overall deformability to diffuse freely but entrapping and immobilizing others with different properties. In vivo, such preferential infiltration of certain vesicle populations into tissues with specific ECM composition or architecture may potentially form a basis for organotropic “homing” of particles.

We have presented experimental data that support and bring together existing computational and theoretical work on particle diffusion through different ECM-like environments. We show that polystyrene beads are a poor model for studying the diffusion of liposomes and that the effect of PEGylation on particle diffusivity is not due to electrostatic effects, as previously claimed, nor through the prevention of adsorption of soluble or colloidal materials on the particle surface, as no such species exist in our system. The design of lipid nanocarriers and engineered liposome-based therapeutics can thus take advantage of nonspecific membrane-matrix interactions to achieve improved penetration and retention in target tissues. Further research on ECM-derived materials and more complex lipid vesicle systems is needed to better understand the diverse molecular interactions that govern the movement of vesicles in the tissue microenvironment.

## Author contributions

R.D. and A.C. designed the project. N.W.T. conducted experiments and wrote the manuscript. OS. analyzed experimental data and provided theoretical derivations. R.D. and A.C. supervised the project and edited the manuscript.

## Supporting material

Document S1. Figures S1–S7, Table S1, and Sections S1–S7

Document S2. Article plus Supporting Material
